# Phenotyping for waterlogging tolerance in crops: current trends and future prospects

**DOI:** 10.1093/jxb/erac243

**Published:** 2022-06-01

**Authors:** Patrick Langan, Villő Bernád, Jason Walsh, Joey Henchy, Mortaza Khodaeiaminjan, Eleni Mangina, Sónia Negrão

**Affiliations:** School of Biology and Environmental Science, University College Dublin, Dublin, Ireland; School of Biology and Environmental Science, University College Dublin, Dublin, Ireland; School of Biology and Environmental Science, University College Dublin, Dublin, Ireland; School of Computer Science and UCD Energy Institute, University College Dublin, Dublin, Ireland; School of Biology and Environmental Science, University College Dublin, Dublin, Ireland; School of Biology and Environmental Science, University College Dublin, Dublin, Ireland; School of Computer Science and UCD Energy Institute, University College Dublin, Dublin, Ireland; School of Biology and Environmental Science, University College Dublin, Dublin, Ireland; Forschungszentrum Jülich, Germany

**Keywords:** Abiotic stress, breeding, flooding, phenotyping, plant imaging, waterlogging

## Abstract

Yield losses to waterlogging are expected to become an increasingly costly and frequent issue in some regions of the world. Despite the extensive work that has been carried out examining the molecular and physiological responses to waterlogging, phenotyping for waterlogging tolerance has proven difficult. This difficulty is largely due to the high variability of waterlogging conditions such as duration, temperature, soil type, and growth stage of the crop. In this review, we highlight use of phenotyping to assess and improve waterlogging tolerance in temperate crop species. We start by outlining the experimental methods that have been utilized to impose waterlogging stress, ranging from highly controlled conditions of hydroponic systems to large-scale screenings in the field. We also describe the phenotyping traits used to assess tolerance ranging from survival rates and visual scoring to precise photosynthetic measurements. Finally, we present an overview of the challenges faced in attempting to improve waterlogging tolerance, the trade-offs associated with phenotyping in controlled conditions, limitations of classic phenotyping methods, and future trends using plant-imaging methods. If effectively utilized to increase crop resilience to changing climates, crop phenotyping has a major role to play in global food security.

## Teaser

Review of waterlogging as a yield limiting stress and the benefits and challenges of applying a modern phenotyping approach to improving waterlogging tolerance of major crops.

## Introduction

The Intergovernmental Panel on Climate Change (IPCC) has predicted increased weather extremes for much of the world’s crop-producing arable regions (Masson-Delmotte et al., 2021). The Food and Agriculture Organization of the United Nations (FAO) forecasted that an increase of about 70% in food production by 2050 is required to meet the demand of an increasing population (FAO, 2021). At the intersection of these monumental challenges is improving crop tolerance to a wide range of stresses exacerbated by more volatile climates. Recent scientific developments have made technologies for genetic analysis increasingly accessible. However, a bottleneck has arisen in the collection of quality phenotypic data and big data analysis to advance crop breeding programmes for stress improvement compared with genetic analysis. Traditional phenotyping methods are labour and time intensive and unsuitable for the large-scale screening of germplasm that is required for breeding purposes. High-throughput phenotyping through technological advances is expected to reduce the labour and time required to obtain phenotypic data while, importantly, increasing the scale of genetic screens, improving reliability of data and the volume of genetic resources that can be investigated.

Water stress is set to be one of the most devastating factors for temperate broad-acre crop yields as many regions are set to encounter more frequent droughts while others are set for regular flooding and waterlogging events. Thus, maintenance of yield under such increased weather extremes is a pivotal objective for future breeding programmes. The mechanisms of and responses to waterlogging have been well studied over the years (Kramer, 1951; Mittra and Stickler, 1961; Grable, 1966; Watson *et al.*, 1976; Trought and Drew, 1980; Jackson and Drew, 1984). Applying the most recent technological advances in genotyping and phenotyping is key to progress from the fundamental groundwork of the past as we aim to limit crop losses to waterlogging. Waterlogging causes an average yield decrease of around a third; however, this impact varies greatly between crop species and with other stress conditions (Tian *et al.*, 2021). Yield reductions range greatly between species, from little reduction in adapted crops such as cultivated rice (*Oryza sativa* L.) to substantial declines in highly sensitive crops such as maize (*Zea mays* L.) (Tian *et al.*, 2020). Waterlogging tolerance may vary greatly within the same species, with valuable genetic resources found in wild relatives and landraces (Zhang *et al.*, 2017). Waterlogging is a highly variable stress as many compounding factors can affect the plant responses, with the duration of the stress period being the most critical of these (Ren *et al.*, 2016; Tian *et al.*, 2021). Also, the growth stage of the plant during which waterlogging occurs can differently affect the final yield (Cannell *et al.*, 1980; de San Celedonio *et al.*, 2014). Changes in soil redox potential greatly alter soil nutrient composition and can lead to elemental toxicity (e.g. iron) or deficiency (e.g. nitrogen) (Bjerre and Schierup, 1985; Huang *et al.*, 2015). Abiotic stress seldom occurs in isolation and combinations of stress can result in compounding effects on yield (Mittler, 2006). The co-occurrence of salinity and waterlogging stresses is increasing worldwide due to intensive irrigation systems, rise of saline water tables, and sea water intrusion (Zeng *et al.*, 2013). Salinity and waterlogging interact in their effects on plant ion relations, growth, and survival as waterlogging causes oxygen deficiency and energy deficits that impair ion transport processes, which are key salinity tolerance mechanisms, resulting in exacerbated effects compared with salinity alone (as reviewed by Barrett-Lennard, 2003; Bennett *et al.*, 2009; Barrett-Lennard and Shabala, 2013).

According to Sasidharan *et al.*, 2017, flooding refers to excessively wet conditions that can be further summarized: submergence or partial submergence occurs when the entire plant is below the water level or when the entire root system and part of the above ground organs are submerged, respectively, whereas waterlogging occurs when the root zone of the crop has become flooded as soil water content reaches saturation while the above ground plant remains above the water level (Sasidharan *et al.*, 2017). The change in medium from air to water greatly reduces the rate of gas diffusion, which in turn upsets the regular balance of nutrient uptake and gas exchange in the rhizosphere (Armstrong, 1980). A range of responses to flooding stress have evolved in the plant kingdom. Aside from rice as the notable exception, most of the world’s important crops are not well-adapted to aquatic or semi-aquatic agriculture. In this review, we focus on waterlogging as it is the most prevailing stress affecting crops across farmers’ fields.

Feeding 9 billion people by 2050 will require major societal changes and increased efficiency at all stages of food production. To address such notable challenges, it is of the utmost importance to maintain yield under stressful conditions, particularly in light of climate change effects. All available measures will need to be taken, from use of best agronomic practices to reduce the effects of waterlogging (Kaur *et al.*, 2020) to the development of tolerant crops. Research and resources will need to be allocated for the improvement of crop resistance to both biotic and abiotic stresses. Studying crop adaptation to stress requires the examination and phenotyping of germplasm in diverse environments, ranging from pot systems to field studies. In this review we aim to assist students and researchers by outlining different phenotyping methods and the challenges associated with them. Here we provide an overview of the challenges faced in adapting crops to waterlogging and the role of plant phenotyping for a sustainable future.

## What are the impacts of waterlogging on plant physiology?

**Fig. 1.**
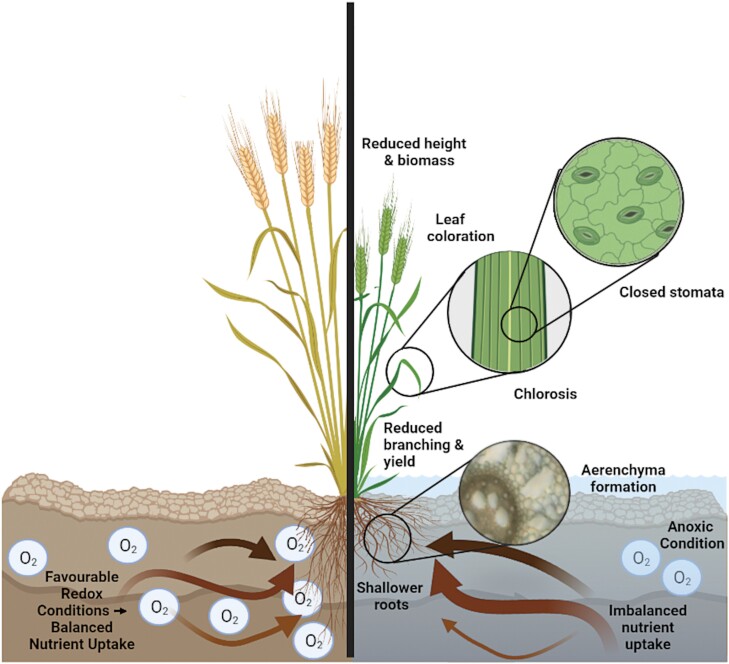
Physiology of waterlogging in a plant. On the left, a barley plant under non-waterlogging conditions in well oxygenated soil. On the right, a barley plant under waterlogging stress in anoxic soil, showing a list of common physiological responses to waterlogging. Barley is used as a hypothetical example, yet these physiological responses are common to other crops experiencing waterlogging. Elements of the figure were created using Biorender.com.

The sessile nature of plants forces them to develop strategies for coping with stresses present in their environment. Waterlogging rapidly reduces oxygen supply to the roots, preventing aerobic respiration and forcing a switch to fermentation for energy (Ashraf, 2012). Fermentation provides only a short-term and less efficient solution to the energy crisis, contributing only a small fraction of the energy produced under control conditions (Gibbs and Greenway, 2003). Under anaerobic fermentation, starch reserves are rapidly depleted and harmful by-products such as alcohols, aldehydes and reactive oxygen species (ROS) are generated (Sairam *et al.*, 2011; Sauter, 2013; Tamang *et al.*, 2014). Such reduced energy production will in turn result in decreased nutrient uptake, growth, and cell maintenance (Herzog *et al.*, 2016). Photosynthetic activity and stomatal conductance decrease (Gomes and Kozlowski, 1980; Malik *et al.*, 2001; Lee *et al.*, 2017; Posso *et al.*, 2018; Zhang *et al.*, 2019) due to reduced chlorophyll degradation, damage of photosystem II, reduced photosynthetic enzyme activity (Amri *et al.*, 2014; Wu *et al.*, 2015; Ploschuk *et al.*, 2018), low nitrogen content (Drew and Sisworo, 1979), and ROS damage (Andrade *et al.*, 2018). ROS are put into action in stress signalling, yet they need to be tightly controlled as their excess can induce oxidative damage to organelles and impact vital cell structures (Ahanger *et al.*, 2018). In addition, when photosynthetic activity is halted, excess light is absorbed leading to accumulation of ROS (Carvalho *et al.*, 2015; Anee *et al.*, 2019). During waterlogging there is an overall shift by the plant from energy use for growth to use for survival, leading to reductions in growth, height, and yield (Collaku and Harrison, 2002; Tian *et al.*, 2020; Tian *et al.*, 2021), which may ultimately lead to death (Smethurst *et al.*, 2005; Tamang *et al.*, 2014). Taken together, waterlogging impacts plant physiology at different levels ranging from photosynthetic effects to lower yield (Fig. 1).

Several factors influence the severity of waterlogging stress including temperature (Trought and Drew, 1982; Lin *et al.*, 2016; Chen *et al.*, 2017), the rhizosphere microbiome (Lin and Sauter, 2018), growth stage (Ren *et al.*, 2016; Wang *et al.*, 2017; Tian *et al.*, 2021), plant species/accession (Perata *et al.*, 1992; Arguello *et al.*, 2016; Tian *et al.*, 2021), stress duration (Masoni *et al.*, 2016; Ren *et al.*, 2016), and soil texture and chemical composition (Boem *et al.*, 1996; Jiménez *et al.*, 2015). At higher temperature plants are more susceptible to waterlogging, as temperature stress on its own causes hormonal imbalance, and a reduction in photosynthetic rate and carbohydrates (Lin *et al.*, 2016; Chen *et al.*, 2017). Waterlogging stress is exacerbated by high temperatures possibly due to an increased oxygen depletion and consumption (Trought and Drew, 1982). Waterlogging impacts plants differently according to their growth stage. For example, in wheat, waterlogging at tillering stage leads to a reduction in spike and grain numbers, while waterlogging at booting stages reduces grain weight (Wu *et al.*, 2015). Waterlogging duration is a crucial determinant of stress severity; for example, maize grain weight reduction after 6 d of waterlogging was about twice as much as after 3 d (Masoni *et al.*, 2016; Ren *et al.*, 2016).

The first effects of waterlogging are experienced in the rhizosphere as microorganisms compete with roots for limited oxygen (Lin and Sauter, 2018). Furthermore, waterlogging affects availability of nutrients in soil (Sharma *et al.*, 2018), causing an imbalance in nutrient uptake of plants and leading to both shortages and toxic build-ups of different plant nutrients (Boem *et al.*, 1996; Jiménez *et al.*, 2015). The nutrient uptake in waterlogged soils is affected by changes in the chemical reduction of some nutrients (notably nitrate, ferric, and manganese ions) due to the anaerobic respiration by soil bacteria (Ponnamperuma, 1972), limited root surface, as well as reduced proton motive force, less negative membrane potential and reduced metabolic control of xylem loading. Waterlogging substantially reduces plant concentrations of nitrogen, phosphorus, potassium, magnesium, copper, zinc, as well as toxicity of iron and manganese (Steffens *et al.*, 2005; Tong *et al.*, 2021). In particular, nitrogen and phosphorus shortages will reduce plant growth and consequently biomass (Zambrosi *et al.*, 2014), causing leaf chlorosis due to the remobilization of nitrogen to new leaves (Drew and Sisworo, 1977). Amelioration of such nutritional effects has been achieved after nitrogen application (Zhou *et al.*, 1997) and phosphorus fertilizer (Pang *et al.*, 2007; Ylivainio *et al.*, 2018).

## How do plants respond to waterlogging?

The low O_2_ status of the rhizosphere under waterlogged conditions prevents root respiration, which in turn impacts oxygen-requiring metabolic processes, causing changes in plant-growth and nutritional status and leading to cell damage and even death (Drew and Sisworo, 1979; Trought and Drew, 1980). Thus, rapid sensing and signalling of stress are vital to allow for adaptation and damage control. Roots are the first organ to sense waterlogging, and hence play a key role in the waterlogging stress response. Once a plant detects waterlogging, its priority is to reinstate the oxygen supply to the roots, which can be achieved by altering its root morphology and anatomy (Pedersen *et al.*, 2021). Under waterlogging conditions and due to lack of oxygen, older roots die, but some species can produce new roots closer to the surface—adventitious roots—which act as an acclimation mechanism to low O_2_ status, facilitating gas exchange (Sauter, 2013; Pedersen *et al.*, 2021). In some species such as rice, some roots can even grow from the shoot area to further reduce the gas diffusion distance (Sauter, 2013; Steffens and Rasmussen, 2016).

The most emblematic physiological response to waterlogging is the development of aerenchyma to enhance the internal supply of O_2_ along the roots (Justin and Armstrong, 1987; Jackson and Armstrong, 1999). Aerenchyma consists of porous spaces in roots that improve gas diffusion and facilitate oxygen transport from above-ground structures to the roots (Colmer, 2003; Loreti *et al.*, 2016). Aerenchyma can form in primary and secondary tissue (Yamauchi *et al.*, 2018). Primary aerenchyma can be lysigenous or schizogenous, where lysigenous aerenchyma is formed by the death and lysis of cortical cells in roots and schizogenous aerenchyma is formed by separation of adjacent cells through differentiation division and/or expansion of cortical cells (with no cell death) (Yamauchi *et al.*, 2018). Secondary aerenchyma develops from phellogen, forming spongy tissue filled with air spaces outside of stem, hypocotyl, and roots (Yamauchi *et al.*, 2018). Rice is an exception because aerenchyma is formed constitutively (i.e. prior to waterlogging stress), promoting cell survival and enabling a faster induction of further aerenchyma formation. Root cortical aerenchyma formation is another morphological adaptation of plants to waterlogging. For example, barley genotypes with higher root cortical aerenchyma produced significantly higher yield under waterlogging (Manik *et al.*, 2022a). In addition, relatively consistent correlation has been reported between adventitious root development and root cortical aerenchyma formation in barley (Manik *et al.*, 2022b). In addition to aerenchyma, radial oxygen loss from the roots can be reduced through the formation of a barrier that prevents oxygen leakage into surrounding soil and enhances O_2_ diffusion to root tips (Abiko *et al.*, 2012; Watanabe *et al.*, 2017; Pedersen *et al.*, 2021). Some crops such as maize and wheat cannot form a radial oxygen loss barrier (Abiko *et al.*, 2012), yet they have developed other structural changes to cope with radial oxygen loss, namely increased cortex-to-stele ratio and smaller surface area to volume, with both strategies promoting a diffusion of O_2_ along the roots to overcome the root energy crisis (Armstrong and Beckett, 1987; Pedersen *et al.*, 2021).

Waterlogging tolerance mechanisms, including signalling pathways, genes and quantitative trait loci associated with tolerance-related traits, have been thoroughly reviewed (Bailey-Serres and Voesenek, 2010; Mancuso and Shabala, 2010; Fukao *et al.*, 2019; Khan *et al.*, 2020; Jia *et al.*, 2021; Tong *et al.*, 2021). Moreover, the crucial role of transcriptional and translational regulations of specific genes in plant adaptation to waterlogging has been reported (Licausi and Perata, 2009). Here we provide a simplified summary of the molecular mechanisms underlying plants’ response to waterlogging. Inducible aerenchyma formation is dependent on ethylene and ROS signalling pathways through the induction of cell death and the development of above-root primordia during adventitious root formation (Jackson and Armstrong, 1999; Mergemann and Sauter, 2000; Steffens and Sauter, 2009; Yamauchi *et al.*, 2018). Waterlogging prevents gases from leaving the roots through the soil, leading to ethylene build-up in the roots (Voesenek and Sasidharan, 2013), a reduction in root growth (Loreti *et al.*, 2016), promotion of auxin biosynthesis (Qi *et al.*, 2019), cell elongation (Schopfer, 2001), and root gravitropism (Joo *et al.*, 2001). Due to the importance of ethylene and ROS in response to waterlogging, several genes that are involved in ROS production have been identified, including *RBOH*, (Sagi and Fluhr, 2006), as well as ethylene response factors (ERFs).

Ethylene response factor group VII (ERFVII) is known to respond to low O_2_ availability through the mediation of the N-degron rule pathway (formerly known as the N-end rule pathway) of targeted proteolysis (for a complete review of the N-degron pathway see Holdsworth *et al.*, 2020). ERFVII transcription factors act as oxygen sensors through the oxidation of the tertiary destabilizing residue cysteine (Gibbs *et al.*, 2011; Licausi *et al.*, 2011). Substrates containing destabilizing residues of the N-degron pathway mediate proteasomal degradation of proteins via specific E3 ligases (Garzon *et al.*, 2007; Holdsworth *et al.*, 2020). The ERFVII are highly conserved in flowering plants (Nakano *et al.*, 2006) and have been reported in Arabidopsis, rice, and poplar, demonstrating the crucial role of this transcription factor family in response to waterlogging (Loreti *et al.*, 2005; Lasanthi-Kudahettige *et al.*, 2007; Kreuzwieser *et al.*, 2009). The most well-known ERFVII genes include those of rice responses to rapid or deep-water flooding, namely submergence 1 (*Sub1A*) and Snorkel *Skl1* and *Skl2*, respectively (Khan *et al.*, 2020). For example, manipulation of ERFs has been shown to improve waterlogging tolerance through the reduced expression of barley N-recognin E3 ligase (*HvPRT6* gene) (Mendiondo *et al.*, 2016).

Our understanding of waterlogging sensing, signalling, and response has been greatly expanded in recent years through exemplary molecular lab work (for reviews see Bailey-Serres and Voesenek, 2010; Zhou *et al.*, 2020). In contrast to drought, waterlogging stress *per se* can be more complex and varied. In fact, there are numerous developmental responses to waterlogging that differ between roots and shoots as well as between species. Crop species will respond differently to waterlogging, and some responses can be considered to be adaptations (e.g. aerenchyma formation) while others can be interpreted as an injury (e.g. chlorosis) (as reviewed by Parent *et al.*, 2008; Zhou, 2010). As a result of such complexity, standardization of waterlogging protocols has proved to be difficult. Instead, a plethora of approaches exist in the literature (e.g. pots in tanks or field trials) across a range of crop species, where numerous traits such as biomass, root porosity, photosynthetic parameters, and chlorophyll content have been assessed (Table 1).

**Table 1.**
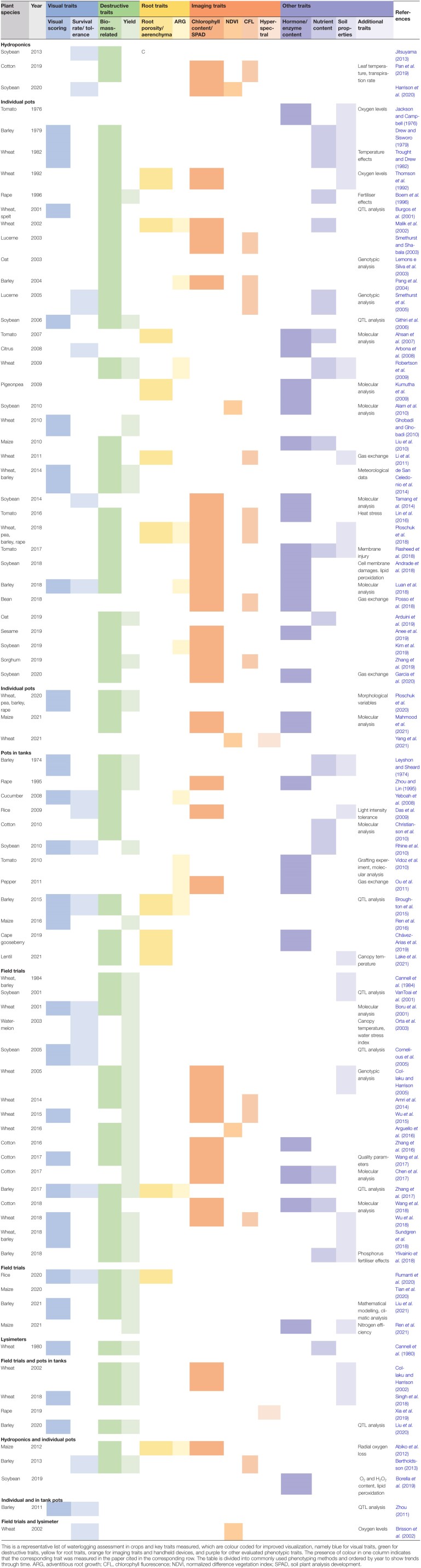
Compilation of crop phenotyping approaches to waterlogging tolerance found in literature

Phenotyping in controlled conditions is typically a straightforward approach to assess crop performance under abiotic stress, including waterlogging, as it simplifies and reduces other confounding effects. Breakthroughs in elucidating the intricacies of waterlogging stress response greatly aid our understanding, but there is a gap between advances in the laboratory and the stress tolerance of crops in the field. Phenotyping for waterlogging in controlled conditions only simulates to some degree what happens in field conditions, due to the intricacies of field trials. Field-based work for waterlogging is easier said than done, due to several challenges ranging from levelling the soil to availability of water sources. Nevertheless, studies have been carried out to bridge the gap between controlled and field conditions (Collaku and Harrison, 2002; Setter *et al.*, 2009; Zou *et al.*, 2014), showing that some traits were significantly correlated between the two settings. We believe that advances in genetic analysis paired with plant phenotyping are pivotal to bridging this gap towards a more food secure future.

## What are the advantages and challenges when phenotyping for waterlogging?

Due to the variability of waterlogging stress and its compounding factors in terms of plant response, there are no standardized phenotyping protocols for screening waterlogging stress. Phenotyping for waterlogging has been achieved using different methods (e.g. pots in tanks or field assays) across a range of crop species while assessing a plethora of traits such as biomass or chlorophyll content (Table 1). Further experimental variation is observed for stress duration, growth stage during stress imposition, and the inclusion of recovery periods (Striker, 2008, 2012; Tian *et al.*, 2021).

Many laboratory-based screenings for waterlogging focus specifically on anoxia to elucidate the mechanisms of hypoxia response. Much work undertaken in model species such as Arabidopsis has been used to expand our knowledge of waterlogging stress. Highly controlled conditions and growth media allow for the isolation of anoxia stress by removing compounding factors found in the field or pots. Laboratory-based methods such as hydroponics, agar, and starch have been used to simulate the hypoxic conditions of waterlogging (Hunt *et al.*, 2002; Bertholdsson, 2013; Miricescu *et al.*, 2021). The use of such methods with a tighter control over the growth media, i.e. hydroponics or individual pots, improves reproducibility and allows for easier harvesting of roots. However, such methods remove the important interaction of soil type and soil toxicity persisting after treatment (Pang *et al.*, 2004; Khabaz-Saberi *et al.*, 2006). In general, laboratory-based phenotyping uses highly controlled environments, either glasshouses or fully controlled growth chambers. Glasshouse experiments offer more controlled conditions at a smaller scale while maintaining fairly similar environmental conditions to the region in which they are located (light intensity, photoperiod, and temperature). Moreover, other abiotic and biotic stresses can be better controlled within glasshouse experiments while providing easily obtained environmental data. Glasshouses can offer varying levels of environmental control ranging from basic slatted-side glasshouses that only provide wind protection (Pang *et al.*, 2004) to highly controlled conditions including use of supplemental lighting, temperature control, and automated watering (Luan *et al.*, 2018). Glasshouse trials also reduce the risk of disease, which may influence phenotypic results. Growth chambers offer further control of environmental factors such as light and temperature at a, once again, minimized scale.

Several waterlogging experiments for physiological and genetic purposes, use individual pots or pots placed in containers, including concrete or plastic tanks (Table 1). Potted experiments allow greater control over soil composition and root architecture (Negrão and Julkowska, 2020). Although potted experiments present a vastly different root environment from field conditions, there are benefits to individual samples (Ploschuk *et al.*, 2018). Roots are more accessible for analysis within potted experiments allowing for better investigation of morphological changes such as aerenchyma or radial oxygen loss barrier formation in response to waterlogging (Rasheed *et al.*, 2018). Allowing for destructive and non-destructive root phenotyping is a key advantage of using pots over field experiments as damage during harvest is reduced, allowing for detailed investigation of root morphology under waterlogging stress (Cannarozzi *et al.*, 2018). In general, waterlogging stress is achieved by reaching ~110–120% field capacity. We recommend estimating the field capacity of the soil in question using a target weight that is maintained throughout the course of the experiment. Duration of stress and recovery period should be optimized based on phenotyping objectives, crop species, growth stage, and experimental setup (Striker, 2008).

The controlled conditions of a pot in a glasshouse or growth chamber provide high resolution of individual sample data at a lower throughput. On the other hand, phenotyping in the field can provide lower resolution data, yet field trials represent crop performance at a more agronomically relevant scale as yield can be assessed (Negrão and Julkowska, 2020). Waterlogging screening in pots containing soil substrate offers conditions closer to the ‘natural’ field environment compared with the very tightly controlled environment found in hydroponics or other substrates (e.g. perlite). The key issue to consider with glasshouse trials is whether the results obtained strongly mirror those of the field. There are many environmental elements in the field that will affect results and that need to be considered when comparing results from the glasshouse. The disparity between glasshouse and field trials is also present when conducting phenotyping.

Screening for waterlogging tolerance in the field comes with a flood of challenges as waterlogging is a highly variable stress. Temperature, pH, and nutrient and mineral quantities will change based on the water source and/or the duration of the waterlogging event. Waterlogging events caused by rainfall are sporadic in field conditions due to differences in soil elevation, compaction, and nutrient heterogeneity, which can introduce significant noise into the data. Careful selection of field site, experimental design, and replication is critical to assess differences between experimental units (i.e. comparing cultivar tolerance). Phenotyping in field conditions for waterlogging tolerance will also need to take such variability into consideration. Ideally, one would prefer to use a naturally waterlogging prone site for repeated trials because artificially waterlogging a field site will require substantial resources as water must be supplied continuously to maintain the flooded conditions. Although field research such as that of Borrego-Benjumea *et al.* (2021) utilizes such sites, the availability of naturally waterlogging prone environments for agronomic, physiological, or genetic experiments is limited and so waterlogging must be artificially simulated. Creating homogeneous and reproducible field conditions for waterlogging is an arduous endeavour with many factors that must be considered such as laser levelling field sites in some cases. Firstly, very few locations will have precipitation reliable enough to guarantee waterlogged conditions and so a water source must be available on site. Secondly, the water source selected is very important as agricultural run-off and mineral contents can add extra factors to the waterlogging stress. In fact, irrigating with enough water to reach field capacity can result in the rapid accumulation of any chemical constituents present in the water source. Thirdly, the duration of stress in field trials is a decision that should not be taken lightly because waterlogging duration is a key factor in determining the damage sustained by the crop. Moreover, the duration of the stress will impact the cost of the experiment as well as the farm environment. The cost of sourcing and pumping water to the experimental site may limit not only the duration of the waterlogging treatment but also the scale of the screening. Finally, waterlogging for the duration of the crop growing season is only possible where water usage and resource availability are less restricted. Waterlogging duration usually ranges from 8 to 28 d for crop species experiments (Striker, 2008). The interaction of waterlogging treatment duration and crop growth stage at stress imposition can have varied effects on final crop yield (Cannell *et al.*, 1980; Belford *et al.*, 1985; de San Celedonio *et al.*, 2014; Tian *et al.*, 2021).

We emphasize the need to perform a pilot or optimization trial to test the soil percolation, water flow rates required, duration of stress, etc. Also, it is important to note that during the stress imposition period, one must monitor the levels of water content in the soil, which can be achieved gravimetrically (i.e. removing soil core samples and obtaining wet and dry weights) or with water and oxygen probe sensors. This is helpful for determining the overall stress resistance among genetic resources, as well as ensuring trackability of changes occurring in different years of field testing. After the considerable effort of experimental setup and the continual waterlogging of the field site has been completed, it is important to ensure that meaningful data are carefully logged, obtained, and analysed.

Classically phenotypic scoring in glasshouse environments (e.g. visual stress scoring) or field environments (e.g. flowering time) has been undertaken by trained labourers; however, this process is tedious and subjective to the assessment of those undertaking the scoring (Negrão and Julkowska, 2020). Furthermore, morphological traits such as height, growth stage, and chlorosis can be recorded, yet such recording is laborious and time-consuming and depends on the scale of the screening and size of the experiment. Technological advances now allow for high-throughput collection of data via imaging sensors mounted on phenotyping platforms for relatively low cost. High-throughput phenotyping utilizes a range of sensors that can be mounted on ground, aerial, or even orbiting platforms. Imaging sensors provide objective data that can be used to track and assess the plant response to stress non-invasively over time, thus offering an unprecedented amount of quantitative data (Negrão and Julkowska, 2020).

## Can image-based methods be used to phenotype for waterlogging stress?

Phenotyping using imaging sensors and platforms continues to advance at an ever-increasing rate, meaning that the bottleneck of phenotypic data collection is eased, and new challenges come to the forefront (Roitsch *et al.*, 2019). Increased spectra, scale of experiments and frequency of imaging all result in higher accuracy and consequently vast amounts of data. It is important that data is utilized to the fullest, ideally by maintenance of open-source databases employing FAIR (findable, available, identifiable, reusable) principles. As mentioned before, standardization of protocols and the reporting of in-depth environmental data and metadata are key to promote the usability of datasets for years to come. Here we introduce image-based phenotyping, highlighting its associated challenges and its promise in assessing waterlogging tolerance.

Imaging sensors can be classified based on the portion of the electromagnetic spectrum they cover. Researchers use visible imaging sensors that cover a wavelength range of 400–700 nm to capture the information lost due to the human eye’s limitations. More commonly known as red, green, blue (RGB) sensors, these visible imaging sensors are cheap to manufacture and it requires little experience to interpret the collected data. Imaging sensors that go beyond the visible wavelength are considered spectral imaging sensors, being categorized by the number of bands and how narrow each band is in a spectral image. If a spectral image has between three and 10 wide bands, it is generally referred to as a multispectral image. However, if the image instead consists of hundreds of bands, then it is referred to as a hyperspectral cube. Spectral imaging sensors are expensive and require extensive knowledge to interpret the results. However, due to its broad application and great potential for stress determination, spectral imaging has been extensively used in crop phenotyping (Beisel *et al.*, 2018; Bruning *et al.*, 2020).

**Fig. 2.**
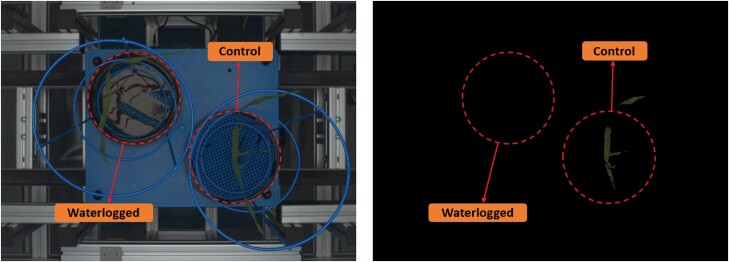
Segmentation of barley plants imaged using a photon system instruments (PSI) imaging suite. (A) Two barley plants (one waterlogged and one control) imaged with a RGB sensor, Sony IMX253LQR-C with a resolution of 4112 × 3006 pixels. (B) Image after application of a colour segmentation method to remove the unwanted noise of the image.

In controlled environments, i.e. glasshouses and growth chambers, imaging sensors can range from affordable low-cost Raspberry Pi cameras to expensive custom-built imaging suites (López-López *et al.*, 2016; Tovar *et al.*, 2018). The use of low-cost equipment was demonstrated by Xia *et al.* (2019), where two imaging sensors, namely RGB and hyperspectral, were used to detect waterlogging stress in oilseed rape. In this study, the authors combined hyperspectral images, ranging from 400 to 1000 nm and targeting 240 channels, with quadratic discriminant analysis, enabling the detection of waterlogging stress in oilseed rape using only six optimal wavelength channels with almost 95% accuracy (Xia *et al.*, 2019). Regardless of cost, imaging sensors still encounter challenges that hinder the quality of the images produced. For example, low-cost devices such as the Raspberry Pi can struggle to handle the impact of image noise. Image noise can be defined as a visual distortion, including variation in brightness, grainy structure, or fluctuations of colours, which can be resolved by an imaging expert. On the other hand, custom-built imaging suites eliminate the influence of noise that may leak onto an image but are expensive and require trained personnel. Image noise can also refer to undesirable objects (e.g. pots, frames, and machinery) that obstruct the image’s main feature— the plant. To remove image noise, a technique called ‘chroma key’ is used to split an image into sub-groups—image segmentation (Agata *et al.*, 2007). In plants, the standard colour blue is used to assist the segmentation process. Segmentation is not easy and is frequently hindered due to challenges relating to the acquisition of each image. Imaging waterlogged plants offers an extra challenge to segmentation as water in the pot reflects the lights of the imaging sensor, reducing the effectiveness of the image segmentation method (Fig. 2). Another challenge to image segmentation is unwanted algal growth. The use of gravel or plastic pellets in the pots to reduce water reflection can help in overcoming the segmentation challenge of waterlogged plants. Furthermore, multidisciplinary research teams comprising computer scientists and computer vision experts are likely to play a substantial role in the improvement of the segmentation process.

The use of artificial intelligence (AI) has increased in popularity in plant stress phenotyping using imaging technologies. One sub-field of AI is machine learning (ML), which is commonly defined as the development of a computer system that can learn from a dataset without following explicit instructions (Mitchell, 1997). ML has considerably changed how the process of image segmentation and stress classification can be studied. The typical use of ML is split based on the learning process into supervised or unsupervised. Supervised learning (e.g. support vector machines, neural networks) requires an annotated dataset (images labelled with stress *vs.* non-stress) to train itself on the classification method. In contrast, unsupervised learning does not require an annotated dataset to learn, but instead uses alternative approaches to analyse data (e.g. clustering). Taken together, supervised learning methods have been shown to produce impressive results in plant stress phenotyping (see review by Singh *et al.*, 2016). This has been demonstrated by Kaneda *et al.* (2017) where both environmental data and RGB images of tomato plants exposed to drought stress were analysed using ML. The authors used a combination of a sliding-window-based support vector regression and a convolutional neural network (both supervised methods) for the accurate prediction of stress traits targeting image data of leaves based on plant wilting motion, demonstrating the advantage of ML with low-cost sensors using low number of images (Kaneda *et al.*, 2017). Unsupervised methods have been advocated for both plant disease classification and segmentation, yet these methods struggle to obtain similar accuracies generated using supervised learning. A new promising computer science trend argues for self-supervised learning (SSL), which is the process of training an algorithm using an unlabelled dataset. This was demonstrated by Nagasubramanian *et al.* (2021, Preprint) where four different types of SSL models were trained and tested on two different plant stress datasets, comprising of biotic and abiotic foliar stresses in soybean plants. Excitingly, SSL significantly improved the data curation process and annotation efficiency for image-based plant stress classification compared with commonly used supervised learning methods.

In field environments, unmanned aerial vehicles (UAVs), wheel-mounted sensors, and satellite imagery are the most popular choices for imaging (Li *et al.*, 2014; Sankaran *et al.*, 2019). The use of ML has been applied to analyse the large influx of data generated from field-based imaging with UAVs. For example, Zhou *et al.* (2021) proposed the use of ML algorithms to quantify the effects of flooding stress on soybeans. In this study, soybean field plots were imaged with multispectral and thermal sensors mounted on a UAV, and supervised learning (feed forward neural network) was used to predict the flooding injury score of each plot imaged (Zhou *et al.*, 2021). Results from this study indicate the effectiveness of ML in estimating the flooding injury score for soybean, demonstrating how field imaging phenotyping and ML could be used to assess waterlogging response in the future. Nevertheless, researchers have also found some success using low-cost handheld imaging devices that do not require expert training, the challenge here being the scale of the research experiment (Rodriguez-Moreno *et al.*, 2017). Handheld imaging devices are limited in both the quality and quantity of the data obtained and can only be efficient for small-scale experiments. Spectral-based imaging sensors present several challenges in the field, including sensor calibration, increased sensor weight, and additional software requirements to analyse spectral images and take into account changes in light due to clouds, sun movement, and shadows. Field image-based phenotyping is subject to a series of challenges due to the influence of external factors. The first challenge is collecting ground-truth data before and during the experiment. Ground-truthing is the acquisition of a value that has been directly observed/measured, and proven true (e.g. flowering time or yield), and is essential to prove or refute the hypothesis of an experiment or statistical model using an imaging method. Thus, the ground-truthing challenge relates to data collection (scoring and harvesting) and the effect of environmental factors on the process (weather data). The second, and most obvious challenge, is data processing. The standard approach to analyse UAV imagery involves transforming many geotagged images into a single georeferenced orthomosaic, providing a bird’s-eye view experiment by stitching many small images together. The stitching process is facilitated by pre-flight geocoordinate collection and flight planning followed by post-flight processing to build an orthomosaic. The orthomosaic is then used for subsequent analysis such as the calculation of vegetation indices (Roberts *et al.*, 2018). In waterlogging field experiments, irrigation equipment such as pipes will act as unwanted objects that will affect further downstream analysis (e.g. commonly used machine learning algorithms). Soil colour changes between waterlogged and control plots will also need to be accounted for during data processing. Due to the nature of saturated soils, aerial imaging is preferred over wheel-mounted platforms to phenotype for waterlogging stress. Also, increased soil evaporation in waterlogged field plots may also complicate thermal imaging analysis due to the introduction of ‘background’ noise.

As previously discussed, the root system plays a critical role in waterlogging responses. Indeed, the plasticity of roots enables plants to change their root system architecture in response to dynamic environmental conditions (Lobet *et al.*, 2019). Root system architecture is defined as the geometric arrangement of structural root features in the three-dimensional soil space (Meister *et al.*, 2014). Due to the difficulties in phenotyping below the soil level, roots are generally less analysed than aboveground organs. To date, classical two-dimensional (2D) techniques such as agar plates or rhizotrons have been used widely to understand root development (e.g. Nagel *et al.*, 2012). Recently, germination/growth pouches have been utilized to study root traits in plants (Acharya *et al.*, 2017; Huang *et al.*, 2020). However, the plant root is a three-dimensional (3D) structure and the results from 2D techniques are often difficult to extrapolate to field conditions (Topp *et al.*, 2013). Currently three tomographic techniques, X-ray computed tomography (CT), magnetic resonance imaging, and positron emission tomography, are applied for 3D phenotyping of roots in soil (see recent reviews by Atkinson *et al.*, 2019; Wasson *et al.*, 2020). Recently, an X-ray CT method was reported to visualize aerenchyma formation in barley roots after 9 d post-waterlogging in 2D and 3D without the requirement for chemical fixation (Kehoe *et al.*, 2022). The X-ray CT method is less destructive, and minimal preparation or fixation time is required. The application of X-ray CT methods can be modified to different plant species for root phenotyping under waterlogging, hence opening new avenues for promising studies.

Several methods have been developed to phenotype different root traits such as crown roots, root surface area (Koyama *et al.*, 2021), or complete root specimens in a soil core (Kücke *et al.*, 1995) and monolith large boxes (Teramoto *et al.*, 2020). The use of ML in root phenotyping has also been demonstrated by putting into action a convolutional neural network- supervised learning method in a field trench (Teramoto and Uga, 2020). Despite the importance of root system architecture in response to waterlogging, there is not a standard root phenotyping methodology to study different aspects of root system architecture under waterlogged conditions. Hence, the imaging research area has much to offer to the research community, and we look forward to seeing several of these root phenotyping methods being optimized for waterlogging studies in crops.

Image-based methods have generated large volumes of data with researchers struggling to handle ‘big data’ and produce the tools needed to mitigate the risks presented by this new data influx. Additionally, generated data (whether image or textual data) must be adequately stored and maintained to remain accessible many years after the experiment is conducted. Thus, data must be based on a data management plan and deposited through an open-source digital repository, with proper ­security measures to certify that it is protected from malicious intent. The goal of any research experiment that handles data (regardless of size) is to: (i) ensure the data can be found; (ii) make the data openly accessible; (iii) make the data interoperable; and (iv) ensure the data are reusable.

By following the FAIR principles, researchers have found various ways to counter big data challenges (Wilkinson *et al.*, 2016). These principles are typically built into the research plan from the beginning of the experiment, simplifying the handling of the increase in volume, complexity, and creation speed of the data. Having a FAIR dataset opens new possibilities for future analysis, ensuring that waterlogging research is reproducible, expediting the development of stress tolerance crops.

## Conclusion

As the effects of the climate crisis begin to increase worldwide, more and more pressure will be applied to food security. Food producers will face new and frequent challenges as weather extremes become more prevalent. Increased waterlogging events are but one of the yield-reducing challenges that will be faced more frequently and intensely. Our ability to maintain yield in the face of these challenges is vital to prevent food shortages. Currently, developing stress resistant cultivars is a slow and laborious process. Combining improvements in genetic analysis and vast amounts of data that can be quickly generated by image-based methods will facilitate the streamlined development and release of resilient cultivars. Yield loss to waterlogging and all future yield-reducing stresses must be counteracted using all available avenues. A combined research effort from the fields of agriculture, biology, genetics, robotics, computer science, and engineering is required.

**Fig. 3.**
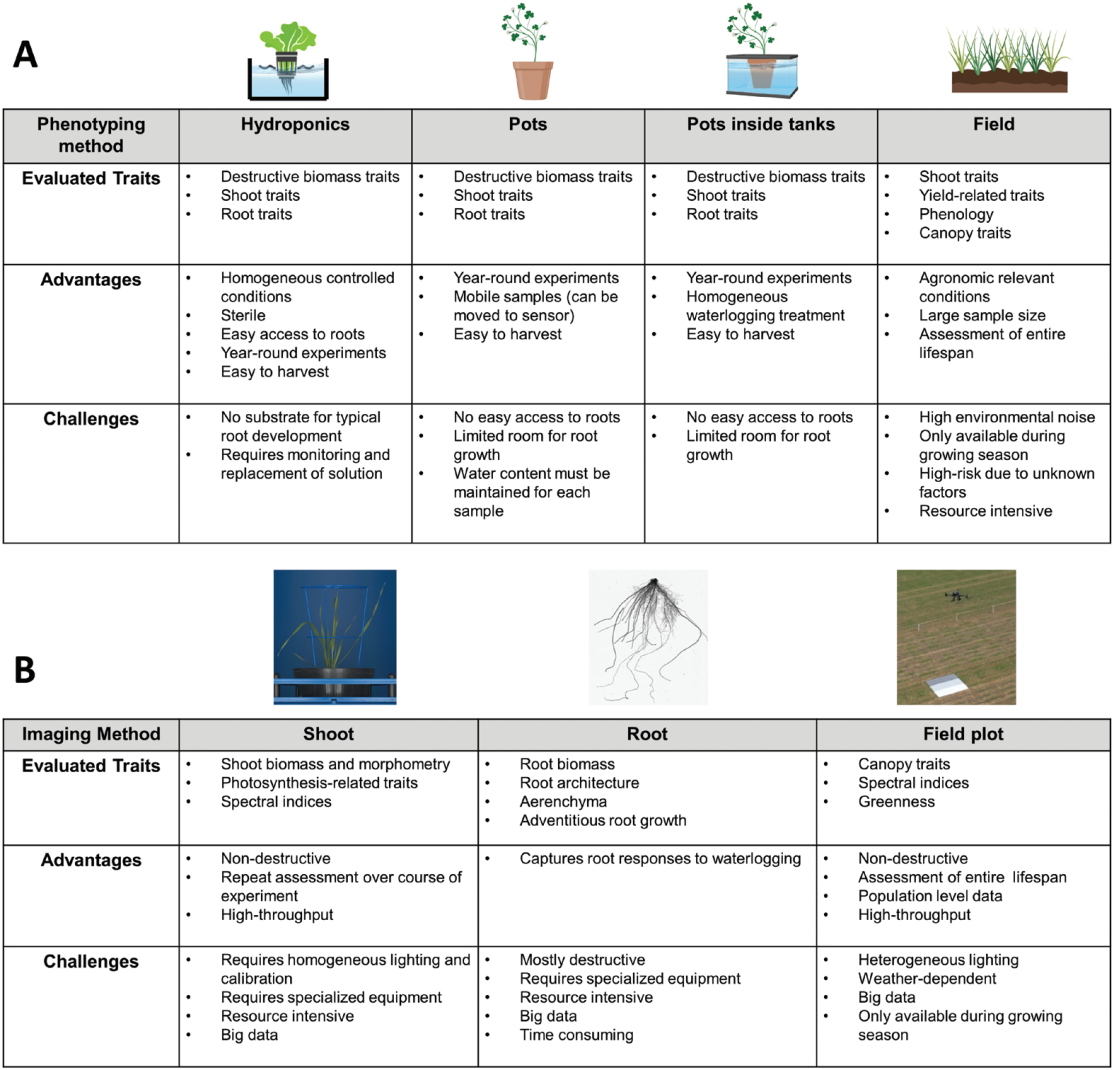
Summary of waterlogging setup systems and phenotyping imaging methods used to evaluate waterlogging stress. (A) Comparison of different waterlogging methods, traits evaluated, advantages, and challenges associated with hydroponics, waterlogging within individual pots, pots within tanks, and waterlogging in field conditions. (B) Comparison of different imaging targets (single plant shoot, single plant root, and plot level canopy) and associated advantages and challenges of each approach using imaging sensors. Figure adapted from (Negrão and Julkowska, 2020). Elements of the figure were created using Biorender.com.

Each waterlogging phenotyping setup (Fig. 3A) and imaging phenotyping method (Fig. 3B) comes with a plethora of advantages and challenges. Thus, the optimal experimental setup should be determined by the research question, target species, available facilities, timeline, and resources (both human and financial). Reproducibility within waterlogging tolerance trials has so far proven elusive (Setter *et al.*, 2009). Combining multiple waterlogging phenotyping methods, for example pots and field, as well as using different imaging sensors will generate extensive datasets, and promises to facilitate and improve the characterization of waterlogging tolerance among crop species. Improved land management and drainage to reduce the prevalence and crop/cultivar selection for waterlogging-tolerant cultivars in high-risk areas will reduce crop losses in the short-term. Unlocking the extensive genetic resources available for all major crops, including landraces and wild relatives, will undoubtedly accelerate waterlogging tolerance. Phenotyping has a large role to play in this progress. From low-throughput image-based systems (e.g. Raspberry Pi) to high-throughput phenotyping in the field (i.e. UAVs), now, like never before, we can monitor the effects of stress on crops non-destructively and through time. Improving tolerance to waterlogging provides unique challenges due to its complex interaction between environment and genotype. Thus, a targeted and proactive phenotyping approach to the challenges ahead will be needed to maintain food security.
